# Seltene anatomische Variante im hepatobiliären System

**DOI:** 10.1007/s00104-023-01886-x

**Published:** 2023-06-05

**Authors:** Viliam Masaryk, Frank Meyer, Uwe Will

**Affiliations:** 1 Klinik für Gastroenterologie, Hepatologie, Diabetologie und Allgemeine Innere Medizin, SRH Wald-Klinikum Gera, Str. des Friedens 122, 07548 Gera, Deutschland; 2 Klinik für Allgemein‑, Viszeral‑, Gefäß- und Transplantationschirurgie, Universitätsklinikum Magdeburg A.ö.R., Leipziger Str. 44, 39120 Magdeburg, Deutschland

## Kasuistik

### Anamnese.

Eine 89-jährige Patientin stellte sich initial in einem auswärtigen Klinikum aufgrund von Oberbauchschmerzen und Fieber vor. Aufgrund des Alters wurde eine konservative Therapie mittels Ceftriaxon bei sonographisch verdickter Gallenblasenwand mit Konkrement und Aerobilie intrahepatisch bei Verdacht auf Cholezystitis mit spontanem Steinabgang initiiert und die Patientin konnte beschwerdefrei entlassen werden. Nach einer Woche wurde die Patientin wieder mit Oberbauchschmerzen, Appetitlosigkeit und Temperaturen bis 40 °C vorstellig. Es bestand das klinische Bild einer Cholangitis.

### Klinische Untersuchung.

Die Patientin zeigte einen Haut- und Sklerenikterus mit abdominellem Schmerzfokus im rechten Oberbauch.

### Diagnostik

**Figure 1:**
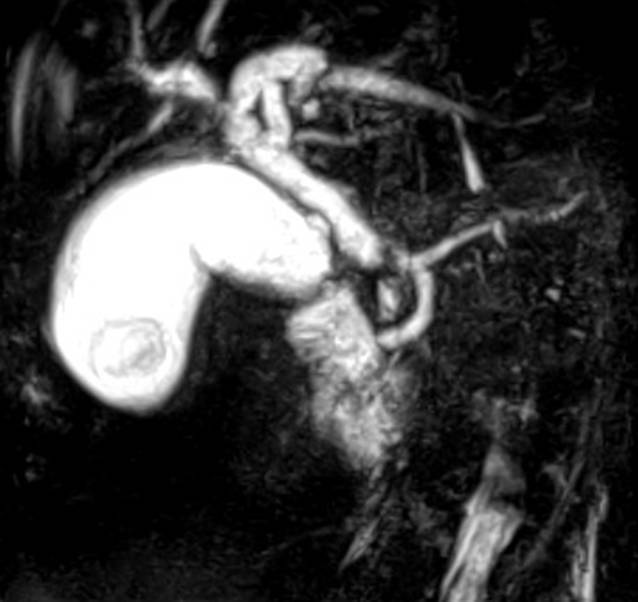

- *Laborchemisch* präsentierten sich: CRP 190 mg/l; ASAT 6,2 μmol/l; ALAT 5,8 μmol/l; AP 7,7 μmol/l; Bilirubin 104 µmol/l; Natrium 117 mmol/l (SI). Lipase und Leukozyten lagen im Normbereich. In der Blutkultur wurde *Enterobacter cloacae* nachgewiesen.
- In der *transabdominellen Sonographie* im auswärtigen Klinikum war der D. hepatocholedochus (DHC) nicht einsehbar, die Gallenblase war hydropisch mit verdickter Wand und einem Konkrement.
- Eine *EUS(endoskopischer Ultraschall)*-Beurteilung des DHC/der Papilla Vateri zur Klärung der Genese des Ikterus war nicht möglich, da die Passage des Pylorus aufgrund eines Ulcus ad pylorum nicht gelang.
- Die *Gastroskopie *erbrachte zwei Ulcera bulbi duodeni Forrest III mit negativem *Helicobacter-pylori*-Status, die Magenschleimhaut sah makroskopisch unauffällig aus.
- Die daraufhin angefertigte *magnetresonanztomographiebasierte Cholangiopankreatikographie* (*MRCP*) erbrachte das Bild eines 19 × 6 mm präpapillären Konkrements mit mäßigem Aufstau des DHC und der intrahepatischen Cholangien sowie einen Gallenblasenhydrops ohne weitere charakteristische Cholezystitiszeichen (Abb. 1).
- In der erneuten *Sonographie *nach der Verlegung ins Klinikum der berichtenden Autoren war der DHC erschwert nur in Linksseitenlage und kurzstreckig einsehbar, auf 9 mm erweitert mit einem echogenen Konkrement. Die Gallenblase war wandverdickt mit einem Konkrement ohne Druckschmerz. Auffällig waren zu diesem Zeitpunkt eine Aerobilie und spontane Luftreflexe in der Gallenblase. Das Pankreas sah unauffällig aus.

**Therapie**. Die Patientin wurde stationär aufgenommen und umgehend eine antibiotische Therapie mit Ampicillin und Sulbactam (Unacid®, Pfizer Pharma GmbH, Berlin, Deutschland) sowie Metronidazol eingeleitet. Aufgrund des MRCP-Befundes wurde die Patientin in die „berichtende“ Klinik verlegt.

**Figure 2:**
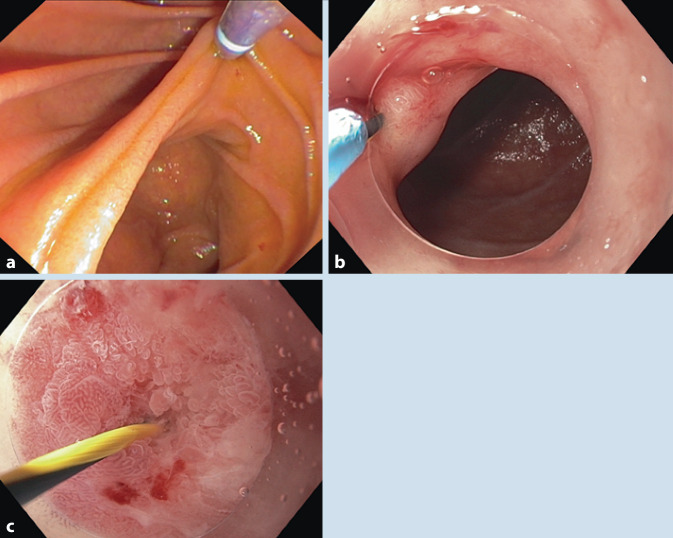

Da die initiale Passage des Duodenoskops zur *endoskopischen retrograden Cholangiopankreatikographie* (*ERCP*) über den Pylorus mit ulzerabedingter Stenosierung und verzogenem und „abgewinkeltem“ Bulbus duodeni nicht gelang, wurde gastroskopisch ein Draht (Wallstent Super Stiff Guidewire, Boston Scientific; Ratingen, Deutschland) transpylorisch platziert. Die Passage des Duodenoskops (TJF-Q180, Olympus; Hamburg, Deutschland) gelang nunmehr drahtgeführt unter fluroskopischer Kontrolle. Im Duodenum fand sich reichlich Galle, die Papilla major war überraschenderweise nicht zu identifizieren (Abb. 2a).

Eine *Re-ERCP* mit diagnostischem Gastroskop (GIF-HQ190; Olympus; Hamburg, Deutschland) mit Aufsatzkappe (Abb. 2b; D‑201, Olympus; Hamburg, Deutschland) unter der Annahme einer atypischen Lokalisation der Papilla Vateri wies ein winziges Ostium intrapylorisch am Übergang vom gastralen zum intestinalen Epithel gegenüber der Ulzera nach (Abb. 2b).

## Wie lautet Ihre Diagnose?

## … Fortsetzung „Therapie“.

**Figure 3:**
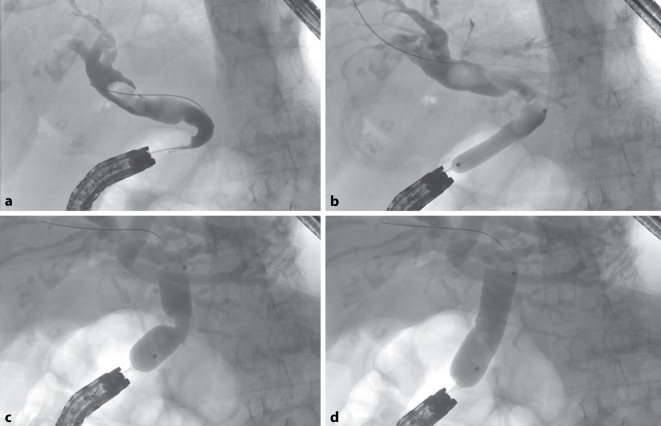

**Figure 4:**
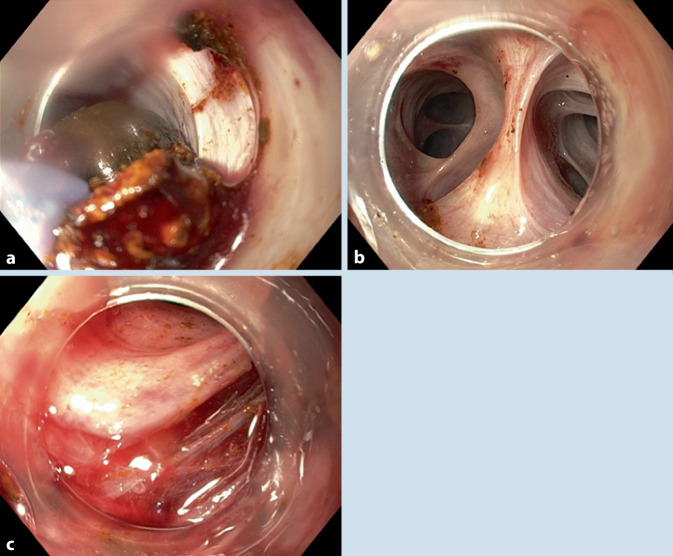

Nach Drahtpassage (Jagwire™, Boston Scientific; Ratingen, Deutschland) und Füllung mit Kontrastmittel (Peritrast®, Dr. Franz Köhler Chemie; Bensheim, Deutschland) war ein dilatierter, „U“-geformter Gallengang mit einer Aussparung des bekannten großen Konkrements zu sehen (Abb. 3a). Der Ductus pancreaticus wurde nicht sondiert und optisch nicht identifiziert. Entsprechend den Literaturempfehlungen wurde eine Ballondilatation ([1–5]; 10 mm, CRE PRO Balloon Dilatation Catheter, Boston Scientific GmbH; Ratingen, Deutschland) statt einer Papillotomie bei aberranter Papillenlokalisation durchgeführt (Abb. 3b). Trotz scharfer Abwinklung passierte ein drahtgeführtes Dormia-Körbchen (Easy-Catch, MTW-Endoskopie W. Haag KG; Wesel, Deutschland) dennoch dieses DHC-Segment. Nach der initialen Dilatation auf 10 mm gelang weder mit Dormia-Korb noch mit „Steinextraktionsballon“ (Extractor Pro XL, Boston Scientific; Ratingen, Deutschland) eine Extraktion des Konkrements. Hiernach wurde sich für eine Ballondilatation auf 15 mm entschieden (Abb. 3c, d). Dies erlaubte direkt nach der Ballondilatation eine direkte orale Cholangioskopie (GIF 190 HQ, Außendurchmesser 9,9 mm, Olympus; Hamburg, Deutschland) mit aufgesetzter Kappe am Gastroskop (Außendurchmesser 12,4 mm; Model D‑201-11804, Olympus, Hamburg, Deutschland) unter CO_2_-Insufflation. Die Steinextraktion erfolgte mittels Dormia-Korb ohne Nutzung von Zerkleinerungsmaßnahmen wie elektrohydraulischer Lithotripsie (EHL; Abb. 4a, b). An der Dilatationsstelle war eine nahezu zirkuläre Schleimhautläsion des DHC mit noch sichtbarer subepithelialer Schicht, aber ohne Kontrastmittelübertritt in die Umgebung sichtbar (Abb. 4c).

### Verlauf

Unter konservativer Therapie mit engmaschiger klinischer Kontrolle kam es zu keinen Komplikationen.

Leukozyten und Bilirubin waren bei Entlassung normwertig, Entzündungsparameter fallend. Eine Cholezystektomie wurde aufgrund des Alters nur bei wiederauftretenden Beschwerden empfohlen.

### Finale Diagnose

Gallensteinbedingter Verschlussikterus mit Cholangitis und atypische intrapylorische Lokalisation der Papilla Vateri bei Ulcera bulbi duodeni Forrest III und Pylorusverziehung inkl. -stenosierung sowie iatrogene gedeckte Verletzung des Gallenganges nach Ballondilatation zur Steinextraktion einer Choledocholithiasis bei Cholezystolithiasis.

## Diskussion

### Hintergrund

Die atypische Lage der Papilla Vateri ist eine sehr seltene anatomische Variante [1–5]. In der Literatur sind nur einzelne Fälle beschrieben. Angegeben werden eine abweichende Lage im Bulbus duodeni oder im aboralen Segment des Duodenums und noch seltener intrapylorisch oder im Antrum. Der eventuell nicht vollständig ausgebildete Papillenapparat erfordert bei papillenassoziierten pathologischen Läsionen sowie therapeutischen Manipulationen besondere Aufmerksamkeit und Expertise (z. B. Perforationsgefahr bei Papillotomie).

### Fallspezifische Aspekte

Bei Verdacht auf eine Cholangitis soll eine mechanische Cholestase gesichert werden. Als bildgebende Diagnostik ist beim Ikterus die abdominelle Sonographie zu präferieren. Die erheblich erschwerte DHC-Darstellung lag am ehesten an der atypischen Lage der Mündungsregion im Pylorusbereich. Bei sonographisch unklarem Befund ist eine weiterführende Diagnostik mittels Endosonographie oder MRT (MRCP) möglich.

Bei schwerer Bulbuspassage ohne vorliegende Stenose können eine vorherige Drahtplatzierung und anschließend eine Passage mit dem Duodenoskop unter zusätzlicher fluoroskopischer Kontrolle hilfreich sein. Auch eine linksseitige Lagerung während der ERCP oder sogar die externe Schienung können die erschwerte Bulbuspassage begünstigen. Sollte das Risiko einer Perforation durch die erschwerte Passage als hoch eingeschätzt werden oder eine ERCP frustran verlaufen, stehen alternative Verfahren zur Verfügung. Alternativ kann z. B. eine perkutan-transhepatische Cholangiodrainage (PTCD) mit 13-Fr-Schleuse, die eine perkutane transhepatische Cholangioskopie mit EHL ermöglicht, eingelegt werden. Auch eine EUS-geführte transhepatische Intervention an den Gallengängen bzw. eine reine EUS-geführte Drainage wären denkbar.

Bei in der Standardposition nicht auffindbarer Papilla Vateri ist an eine sehr seltene atypische Lage zu denken. Angegeben werden am häufigsten eine Lage im Bulbus duodeni oder im aboralen Teil des Duodenums und seltener intrapylorisch oder im Antrum. Ursächlich ist eine atypische embryonale Anlage bei Ausbildung des DHC. Peng et al. untersuchten die ERCP-Befunde von 6113 Patienten, von denen 8 Patienten (0,12 %) eine atypische duodenale Lage hatten [4]. Die intrapylorische Lage ist noch seltener, in der Literatur sind lediglich einzelne Fälle beschrieben.

Der Sphincter Oddi mit Sphinkterapparat kann bei der ektopischen Lage nicht vollständig entwickelt sein, was zu einem inkompletten Verschluss der Papille führen kann. Dies könnte auch die im Verlauf aufgetretene spontane Aerobilie in der Leber und Gallenblase bei der Patientin erklären. Auch ein spontaner Steinabgang oder die Entwicklung einer Fistel zum Magen/Duodenum im Rahmen einer Cholezystitis können zur spontanen Aerobilie führen. Darüber, ob bei der Patientin lebenslang eine Aerobilie bestand, lässt sich nur spekulieren. Zusätzlich wird die „Hook“-Form des DHC als ursächlich für eine Cholestase mit Entwicklung von Konkrementen diskutiert. Der Pankreasgang mündet neben dem DHC und wurde in einigen Arbeiten mit einem separaten Ostium identifiziert. Die intrapylorische Lage wird in der Literatur mit gleichzeitig bestehenden Ulcera ad pylorum [5] wie im vorliegenden Fall beschrieben.

Ein Gastroskop bietet für eine Intervention am Pylorus eine bessere Einstellung des Gallengangs. Eine Papillotomie soll aufgrund des erhöhten Risikos für eine Perforation vermieden werden, eine Ballondilatation ist zu präferieren. Der maximale Durchmesser des Ballons wird je nach Weite des Ganges und Größe des Konkrementes bestimmt, um eine Perforation zu vermeiden. Durch den initial gewählten Ballon von 10 mm ließ sich das Konkrement nicht extrahieren. Die Ballondilatation auf 15 mm führte zu einem nahezu zirkulären Schaden am DHC mit noch sichtbar intakter tiefer (subepithelialer) Schicht. Solche Läsion des Gallenganges ohne Kontrastaustritt ist unter engmaschiger klinischer Observation auch konservativ behandelbar, wie erfolgreich gehandhabt. Auf eine Stentimplantation wurde verzichtet, da es zu keinem Kontrastmittelaustritt kam und ein Stent mit einem größeren Durchmesser als 15 mm notwendig gewesen wäre. Retrospektiv lässt sich mutmaßen, dass eine Dilatation auf 12 mm mit geringerem Schaden am DHC assoziiert und ausreichend für die Steinextraktion gewesen wäre.

**Diagnose:** Abnorme anatomische Lokalisation der Papilla Vateri im Pyloruskanal

Die Cholangioskopie wurde direkt nach der Ballondilatation noch mit aufliegender Aufsatzkappe auf dem Gastroskop angeschlossen. Um eine Verletzung des Gallenganges oder Verlust der Kappe im DHC zu vermeiden, wäre eine Cholangioskopie ohne Aufsatzkappe und mit einem dünnerem Gerät (Kindergastroskop mit Außendurchmesser von 5,5 mm) zu favorisieren. Das Instrumentarium für ein Kindergastroskop beinhaltet sowohl kleine Dormia-Körbchen als auch eine EHL-Sonde. Auch ist kritisch anzumerken, dass die Cholangioskopie unter CO_2_-Insufflation durchgeführt wurde – eine Spülung und Füllung des Ganges mit Kochsalz minimiert das Risiko einer Luftembolie, die auch unter CO_2_-Insufflation vorkommen kann.

Eine Choledocholithiasis bei Cholezystolithiasis stellt eine Indikation zur Cholezystektomie dar. Unter angemessener Nutzen-Risiko-Abwägung bei hochbetagter Patientin erscheint auch ein abwartendes Verhalten trotz der beschriebenen asymptomatischen Wandverdickung und in Anbetracht der geäußerten eher abwartenden Patientenmeinung vertretbar.

### Stärken

Der vorgestellte seltene Einzelfall beschreibt anschaulich die seltene aberrante Lage der Papilla Vateri, die der mit einer hepatobiliären Intervention bzw. Operation konfrontierte Viszeralmediziner (Gastroenterologie, GI-Endoskopist, Viszeralchirurg) kennen sollte hinsichtlich eines adäquaten diagnostischen und therapeutischen Managements.

### Limitation

Es handelt sich um einen sehr seltenen Einzelkasus, dessen Fallkonstellation nur eine begrenzte Aussicht auf verallgemeinernde Schlussfolgerungen auch nur begrenzt durch eine vorgenommene Literaturrecherche zulässt.

## Fazit

Mit der medizinisch-wissenschaftlichen Kasuistik („case report“) sollte, basierend auf gewonnenen klinisch-perioperativen/‑interventionellen Managementerfahrungen und einschlägigen selektiven Referenzen der Literatur, der interessante und selten beschriebene, daher berichtenswerte Fall einer Patientin mit erfolgreicher Behandlung einer Cholangitis im Rahmen einer interventionell-endoskopischen Steinextraktion mit direkter Cholangioskopie nach Ballondilatation bei atypischer intrapylorischer Lage der Papilla Vateri dargestellt werden. Alternativ stehen bei frustraner ERCP auch die PTCD oder die mittels EUS geführte biliäre Intervention zur Verfügung.

Bei nicht in der Standardposition auffindbarer Papille ist an eine sehr seltene atypische Lage zu denken. Angegeben werden am häufigsten eine Lage im Bulbus duodeni oder im aboralen Segment des Duodenums und seltener intrapylorisch oder im Antrum. Ein Gastroskop bietet für die letzteren eine deutlich bessere Einstellung.

Eine Papillotomie soll vermieden werden, die Ballondilatation ist zu präferieren.
